# Allele specific synthetic lethality between *priC *and *dnaA*^ts ^alleles at the permissive temperature of 30°C in *E. coli *K-12

**DOI:** 10.1186/1471-2180-4-47

**Published:** 2004-12-08

**Authors:** Tania Hinds, Steven J Sandler

**Affiliations:** 1 203 Morrill Science Center IVN, Department of Microbiology, University of Massachusetts, Amherst, MA 01003, USA

## Abstract

**Background:**

DnaA is an essential protein in the regulation and initiation of DNA replication in many bacteria. It forms a protein-DNA complex at *oriC *to which DnaC loads DnaB. DNA replication forks initiated at *oriC *by DnaA can collapse on route to the terminus for a variety of reasons. PriA, PriB, PriC, DnaT, Rep and DnaC form multiple pathways to restart repaired replication forks. *DnaC809 *and *dnaC809*,*820 *are suppressors of *priA2::kan *mutant phenotypes. The former requires PriC and Rep while the latter is independent of them. *RnhA339::cat *mutations allow DnaA-independent initiation of DNA replication.

**Results:**

It is shown herein that a *priC303::kan *mutation is synthetically lethal with either a *dnaA46 *or *dnaA508 *temperature sensitive mutation at the permissive temperature of 30°C. The *priC*-*dnaA *lethality is specific for the *dnaA *allele. The *priC303::kan *mutant was viable when placed in combination with either *dnaA5*, *dnaA167*, *dnaA204 *or *dnaA602*. The *priC*-*dnaA508 *and *priC*-*dnaA46 *lethality could be suppressed by *rnhA339::cat*. The *priC*-*dnaA508 *lethality could be suppressed by a *dnaC809*,*820 *mutation, but not *dnaC809*. Neither of the *dnaC *mutations could suppress the *priC*-*dnaA46 *lethality.

**Conclusions:**

A hitherto unknown function for either DnaA in replication restart or PriC in initiation of DNA replication that occurs in certain *dnaA *temperature sensitive mutant strains at the permissive temperature of 30°C has been documented. Models considering roles for PriC during initiation of DNA replication and roles for DnaA in replication restart were tested and found not to decisively explain the data. Other roles of *dnaA *in transcription and nucleoid structure are additionally considered.

## Background

The loading of the DnaB replicative helicase at the *E. coli *origin of DNA replication (*oriC*) is a highly regulated and is thought to be a key step in the assembly of the replisome. DnaB makes important contacts with the τ-subunit of DNA Polymerase III Holoenzyme and DNA primase [1]. DnaB loading at *oriC *during initiation of DNA replication is a sequence specific, cell cycle regulated event dependent on the DnaA and DnaC proteins (reviewed in [2-4]). *In vitro*, in the presence of several other accessory proteins (*i.e. *RNA polymerase, DNA gyrase, HU protein), multiple DnaA proteins bind to four asymmetric 9 bp DnaA binding sites in the 225 bp *oriC *region allowing formation of a protein/DNA complex [5-7]. This in turn causes melting of a nearby AT rich sequence. A complex of DnaB_6_-DnaC_6 _then interacts with the DnaA/*oriC *complex to load DnaB at the AT rich sequence.

It is thought that DnaA may have other roles in the cells in addition to initiation. These additional roles stem from the fact that there are many DnaA binding sites in the chromosome outside the *oriC *region [8,9] and that DnaA binding to these asymmetric 9 bp sites, can bend the DNA [10]. It can be hypothesized based on large number of potential DnaA binding sites in the chromosome and the ability of DnaA to bend DNA that it may influence the structure of nucleoid. It has been shown that if a DnaA binding site falls within a promoter region that mutations in *dnaA *can affect the level of transcription from that promoter [11-14]. Thus, mutations in *dnaA *may have global effects in gene expression and nucleoid structure as well as affecting initiation of DNA replication at *oriC*.

In *E. coli*, *dnaA *is an essential gene. Several different *dnaA *temperature sensitive mutant alleles have been isolated. Many of these share the property that they are double mutants (*dnaA5*, *dnaA46*, *dnaA508 *and *dnaA602 *– Table 1 and [15]). Several of the double mutants share a mutation: a change at codon 184 that replaces an alanine with a valine. Additionally, a *dnaA850::*Tn*10 *mutant has been isolated. This is only viable in strains that have an alternate, *oriC*-independent method of initiation of DNA replication [16].

**Figure 1:**
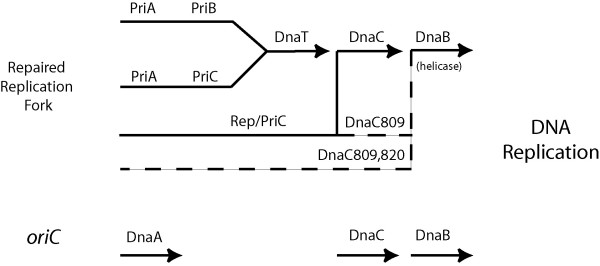
This diagram compares the ways in which the replicative helicase can be loaded either from *oriC *or a repaired replication fork in *E. coli*. Left side of the diagram indicates the starting substrate to which the replisome is to be loaded. The horizontal arrows indicate the way in which the proteins may interact to load the replisome. The dotted lines represent suppressor pathways.

The loading of DnaB by DnaC in *E. coli *can occur away from *oriC *at a repaired replication fork. This is governed by the Replication Restart Proteins (RRPs): PriA, PriB, PriC, DnaT and Rep. The genes coding for these products form multiple pathways to identify the proper substrate and then help DnaC load DnaB (Figure 1 and [17]). Since replication restart is thought to be an essential process, the poor viability (versus complete inviability) of *priA *mutants suggested the availability of alternate pathways. The PriA-independent pathway depends on PriC and Rep [17]. Two types of *priA *suppressor mutations have been found and both map to the *dnaC *gene. The first typified by *dnaC809 *(E176G) and is dependent on the genes in the PriA-independent pathway of replication restart, *rep *and *priC *[17]. The second type of *priA *suppressor has an additional mutation (K178N) in *dnaC *relative to *dnaC809 *and makes this protein's suppression of *priA *mutant phenotypes independent of *priC *and *rep*. This *dnaC *allele is called *dnaC809*,*820 *[17,18]. The multiplicity of replication restart pathways may be a general property in *Bacteria *since *Bacillus subtilis *also has a similar arrangement of PriA-dependent and PriA-independent pathways [19,20].

As mentioned above, DnaA-dependent initiation of DNA replication at *oriC *and replication restart share several properties. The most important of these is that both strive to make protein-DNA complexes that are recognized by the DnaC protein so that DnaB can be loaded. Previous work by Kogoma and colleagues showed that *oriC*-DnaA-independent initiation of DNA replication could take place in an *rnhA *mutant strain [16,21]. This type of initiation of DNA replication was termed Constitutive Stable DNA Replication (cSDR) and is dependent on both recombination and replication restart functions (reviewed in [22] and Sandler, submitted).

To begin testing the roles of *priB *and *priC *in cSDR (reviewed in [22]) required the construction of *dnaA*^ts^*rnhA priB *and *dnaA*^ts^*rnhA priC *triple mutant strains. However when trying to construct these strains, we found that *priC *was required for growth in two different *dnaA*^ts ^strains, *dnaA46 *and *dnaA508*, at the permissive temperature of 30°C. When *priC303::kan *was tested with other *dnaA*^ts ^alleles, the synthetic lethality was found to be allele specific. Two different mutations were found to suppress the *priC*-*dnaA *lethality. One was *rnhA339::cat*, a non-allele specific suppressor of *dnaA *mutants. The other was *dnaC809*,*820*, a suppressor of *priC *and *priA *mutations. The first type suppressed both the *dnaA508*-*priC *and *dnaA46*-*priC *lethality while the latter only suppressed the *dnaA508*-*priC *lethality. These studies suggest that *priC *may have a role in initiation of DNA replication at *oriC *with certain *dnaA *alleles and or that *dnaA *may have an additional role in the cell important for replication restart.

## Results

### *PriC*, but not *priB*, is required for growth in a *dnaA508 *mutant at the permissive temperature of 30°C

**Figure 2:**
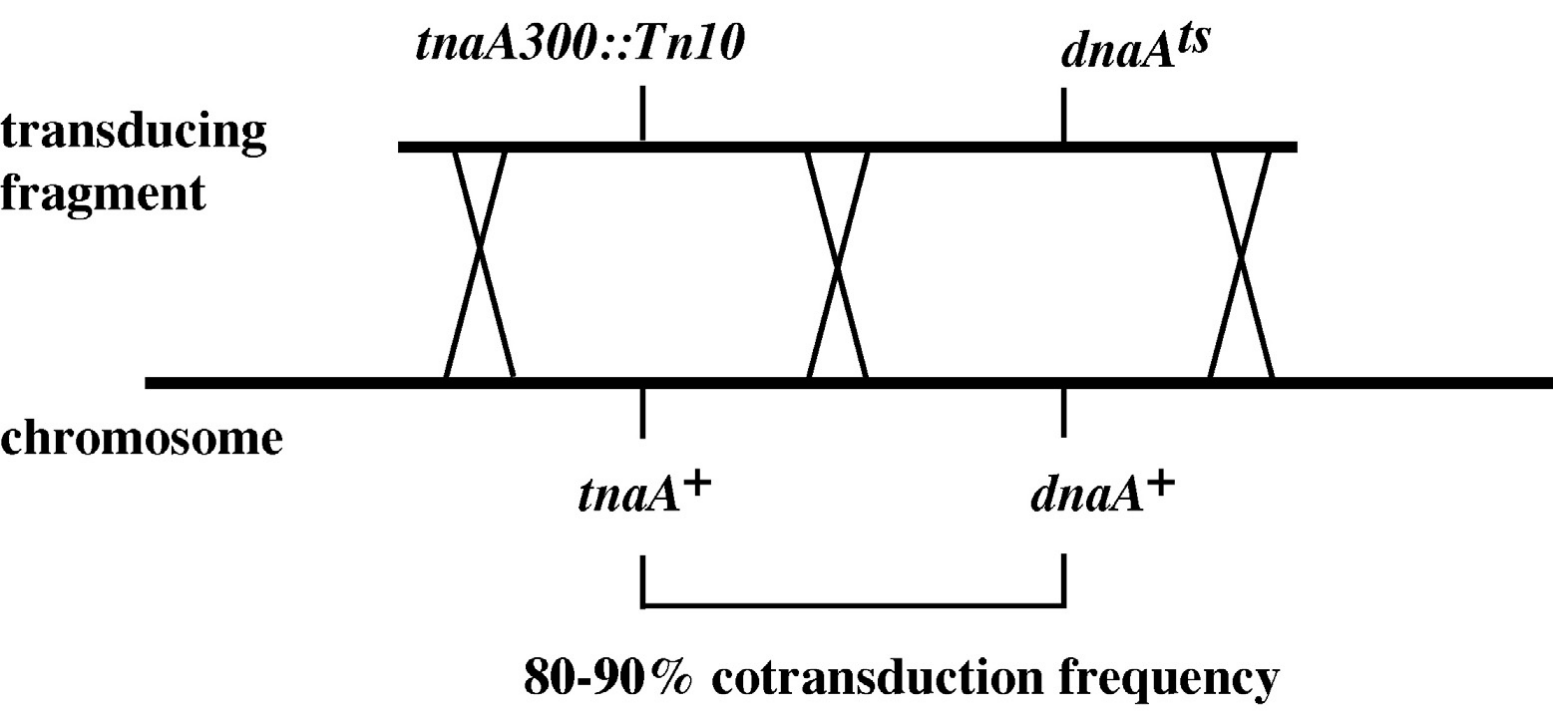
The *tnaA*- *dnaA *region of the *E. coli *chromosome is diagramed on the lower line. The upper line is symbolic of the DNA introduced by the P1 transduction in the standard cross described in this paper where a *tnaA300::*Tn*10 dnaA*^ts ^donor is introduced to a *dnaA*^+ ^recipient. Potential crossover events between the two markers are shown.

We began this study by asking if *priB *and *priC *are required for cSDR. During this study it was found that certain *dnaA*^ts ^could be introduced into a strain containing a *priB *mutation, but not into a strain containing a *priC *mutation. The standard P1 transductional cross used for the introduction of the *dnaA*^ts ^mutant alleles is shown in Figure 2. Table 2 shows that the co-transduction frequency between *tnaA300::*Tn*10 *and *dnaA508 *in a wild type strain is 92% (49/53). Using a *priB *mutant strain as the recipient, the co-transduction frequency between *tnaA300::Tn10 *and *dnaA508 *was approximately the same as when the wild type strain was used as the recipient (data not shown). Surprisingly, when a *priC *mutant was used as a recipient, the co-transduction frequency was 0/72 or 0% (Table 2). This suggested that the *priC303::kan *and *dnaA508 *mutation may be synthetically lethal at the permissive temperature of 30°C. It is also formally possible that *priC303::kan *suppressed the temperature sensitive nature of *dnaA508*. These two possibilities are tested below.

Since it is known that the absence of other gene products (i.e., *rnhA *[23] and *trxA *[24]) can suppress the essentiality of *dnaA*, it is possible that the absence of *priC *might also suppress the temperature sensitivity of the *dnaA*^ts ^allele. If so, then one should be able to detect the presence of the *dnaA *mutation on the chromosome of the 42°C resistant transductants. To test this possibility, Tet^R ^transductants selected at 30°C were screened for a 42°C ^R ^phenotype. These were then further screened for the presence of a restriction site polymorphism (a *Eco *NI site) created by the *dnaA508 *mutation. To do this, the *dnaA *region from the 42°C ^R ^Tet^R ^transductants was amplified by standard Polymerase Chain Reaction methods using the primers, prSJS480 and prSJS481 (Table 3). The amplified DNA was then restricted with *Eco *NI. Examination of eight independent 42°C^R ^Tet^R ^transductants, constructed using JC12390 as the donor, revealed no case in which a restriction pattern was consistent with the presence of the temperature sensitive allele (data not shown). From these results, it is concluded that the *priC303::kan *mutation does not suppress the absence of *dnaA508 *and is synthetically lethal with it.

### *PriC303::kan *is synthetically lethal with *dnaA46 *and *dnaA508*, but not with *dnaA5*, *dnaA167*, *dnaA204 or dnaA602*

It is possible that either the *dnaA508*-*priC303::kan *synthetic lethality at the permissive temperature of 30°C is allele specific or occurs with all *dnaA*^ts ^alleles. To distinguish between these two possibilities, several other *dnaA*^ts ^alleles were tested. Selection of a diverse collection of mutations to test was aided by an already large repertoire of characterized *dnaA*^ts ^alleles [15] and the recent elucidation of the crystal structure of DnaA from *Aquifex aeolicus *[25]. This allowed the selection of several temperature sensitive *dnaA *alleles that had different amino acids substitutions in different parts of the protein (Table 1). Hence, it was attempted to introduce *dnaA5*, *dnaA46*, *dnaA167*, *dnaA204 *and *dnaA602 *into the *priC303::kan *strain (SS145) using the selectable marker *tnaA300::Tn10 *as before. Table 2 shows that the synthetic lethality only occurred additionally with *dnaA46*, but not with *dnaA5*, *dnaA167*, *dnaA204 *or *dnaA602*. It is concluded that the synthetic lethality between *priC303::kan *and *dnaA508 *and *dnaA46 *at 30°C is allele specific.

### The *priC303::kan dnaA*^ts ^synthetic lethality is solely due to the absence of only *priC *and the presence of the *dnaA*^ts ^mutation

Since both the *dnaA *and *priC *genes are in operons, it is possible that the synthetic lethality seen above is due to not just the mutation in *priC *or *dnaA*, but also due to that mutation and or polar effects on downstream genes within their respective operons. While the potential polar effects of a *priC303::kan *insertion mutation are easily envisioned, the potential polar effects of a *dnaA *missense mutation are less obvious. This is tested here as well because, as introduced above and discussed below, *dnaA *mutations can have effects on the level of transcription of promoters in which there are DnaA binding sites. Since *dnaA *binds to its own promoter and autoregulates its own expression [12,26], it is possible that *dnaA *mutation may effect transcription from its own promoter and subsequently effect *dnaN *and *recF *expression.

It was first tested if the synthetic lethality between the *dnaA508 *and *priC303::kan *was dependent solely on the *priC *gene. This was necessary to determine because *priC303::kan *is an insertion mutation and could be polar on the downstream gene, *ybaM*. This was tested by cloning *priC *into a plasmid (pTH1, see Methods) and seeing if the *priC *plasmid could complement the synthetically lethal phenotype. pTH1, containing just the *priC *promoter and gene, in the *priC303::kan *mutant strain (SS145), allowed the *dnaA508 *allele to be introduced into that strain at the wild type co-transduction frequency of 90% (data not shown).

It was then tested if the synthetic lethality was due to polar effects of *dnaA508 *or *dnaA46 *on downstream *dnaN*-*recF *expression. This was tested in a similar way. A plasmid, pAB3 [27], that expresses the *dnaA *gene *in trans *was introduced into the *priC303::kan *mutant strain (SS145). This strain was then used as a recipient in a cross with either ALO450 (*dnaA46 tnaA300::*Tn*10*) or JC12390 (*dnaA508 tnaA300::*Tn*10*). Tet^R ^transductants were selected at 30°C. In each case, several transductants were selected and screened for the presence of the *dnaA*^ts ^mutation by backcrosses to JC13509. The temperature sensitive phenotype associated with *dnaA508 *and *dnaA46 *was detected in each case (data not shown).

It is concluded that the synthetic lethality seen between *priC303::kan *and *dnaA508 *or *dnaA46 *is due to solely the absence of *priC *and the presence of the *dnaA*^ts ^mutation.

### *RnhA *mutations suppress the *priC-dnaA *synthetic lethality

It has been shown that *rnhA *mutations are non-allele specific suppressors of both *dnaA*^ts ^and *dnaA *insertion mutations [16]. The mechanism of suppression is thought to be the stabilization of R-loops on the chromosome [22]. To determine if the *priC*-*dnaA *synthetic lethality is suppressed by a mutation in *rnhA*, it was attempted to introduce *dnaA508 *and *dnaA46 *into a *priC303::kan rnhA339::cat *(SS1531) double mutant strain. It was found that this *dnaA*^ts ^*rnhA priC *triple mutant combination was viable in each case (see SS1543 and SS3032 in Table 4). This suggested that the cause of the *priC*-*dnaA *lethality was a defect in the mutant *dnaA *protein's ability to initiation of DNA replication and that *priC *has some role in initiation of DNA replication in the *dnaA508 *and *dnaA46 *mutant strains.

### *DnaC809*,*820*, but not *dnaC809*, suppresses the absence of *priC *in the *dnaA508 *mutant at 30°C

The above experiment suggested that PriC has a role in initiation of DNA replication in certain *dnaA *mutants. If so, then suppressors of *priC*'s role in replication restart should not suppress the *priC*-*dnaA *synthetic lethality. Two types of replication restart suppressor mutations are known and were tested [18,28]. *DnaC809 *suppresses the phenotypes of *priA2::kan *and *dnaT82*2 [28,29]. *In vitro*, DnaC809 can suppress the absence of all RRPs on several different substrates [30]. *In vivo *however, *priA2::kan *suppression requires *priC *and *rep *[17]. *DnaC809 *can be additionally mutated to make the *priA *suppression both *priC *and *rep *independent [17]. This additionally mutated *dnaC *allele is called *dnaC809,820 *[18]. To test the above hypothesis, *priC303::kan dnaC809 *(SS1099) and *priC303::kan dnaC809,820 *(SS1100) strains were constructed (Table 4) and used as recipients in crosses with the donor P1 from either JC12390 (*dnaA508*) or AL0450 (*dnaA46*). Table 2 shows that when *dnaC809*,*820 *was used as the recipient and JC12390 as the donor that 41/48 or 83% of the Tet^R ^transductants were also temperature sensitive (they inherited the *dnaA508 *allele). However, when only *dnaC809 *was used as the recipient, 0/63 Tet^R ^transductants inherited the temperature sensitive phenotype. It is concluded that *dnaC809*,*820 *can suppress the absence of *priC *in the *dnaA508 *mutant and *dnaC809 *cannot. The *dnaA46 *allele was additionally tested and was not suppressed by either *dnaC809 *or *dnaC809*,*820 *(Table 3). From this it is concluded that *dnaC809*,*820 *is able to suppress the absence of some *dnaA*^ts ^allele. In contradiction to the suggestion of the above *rnhA *experiment, this result suggests that *dnaA *may have some role in replication restart necessary in a *priC *mutant.

## Discussion

This paper shows that *priC*, a gene involved in both the PriA-dependent and PriA-independent pathways for replication restart, is also required for cell viability in two of six *dnaA*^ts ^mutants at the permissive growth temperature of 30°C. These results were surprising on at least two accounts. The first is that *in vitro *systems for either replication restart or initiation of DNA replication at *oriC *posed no requirement for the DnaA or PriC protein respectively. The second is that *priC *has no known role *in vivo *in initiation of DNA replication (the only reported role is in replication restart [17]) and that *dnaA *has no known role in replication restart.

One way to answer the question of why the mutations are synthetically lethal is to see what types of mutations may suppress the lethality. *RnhA339::cat*, a non-allele specific suppressor of *dnaA *mutants role in initiation of DNA replication, could suppress the *priC*-*dnaA *synthetic lethality for both *dnaA*^ts ^mutant alleles. Such suppression is strong evidence that *priC *and *dnaA *may both be missing a function needed during initiation of DNA replication. It was further observed, however, that *dnaC809*,*820 *(but not *dnaC809*) could suppress the absence of *priC *in the *dnaA508 *mutant. Neither *dnaC809 *nor *dnaC809*,*820 *could suppress the *dnaA46*-*priC *synthetic lethality. *DnaC809*,*820 *is a PriC-independent suppressor of several genes required in replication restart. This suppression implicates *dnaA *in replication restart. Thus the inferences from the two types of suppressors seem to contradict one another.

What function(s) in *dnaA*^ts ^strains are missing for initiation of DNA replication that make the strain dependent on *priC *at 30°C? A structure-function analysis of DnaA would help to answer this question. Briefly, based on alignments of DnaA proteins, the X-ray crystal structure of the DnaA protein from *Aquifex aeolicus *and much research on the genetics and biochemistry of DnaA, the DnaA protein can be divided into four domains with four proposed functions: Domain I) DnaB recruitment, Domain II) Linker region, Domain III) ATP binding and Domain IV) DNA binding [25,35,36]. Table 1 shows that the six mutations tested substitute amino acids spread throughout DnaA. The two mutants that show a requirement for *priC *have mutations in Domains I (DnaB recruitment) and III (ATP hydrolysis). However, several of the mutations not requiring *priC *also affect Domain III. An interesting aspect to *dnaA *genetics is that many temperature sensitive mutants have two mutations (Table 1 and [2]). The *dnaA5*, *dnaA46 *and *dnaA602 *all have mutations in Domain III near the ATP binding region. Their second mutations cause amino acid replacements in other domains. Unfortunately, the positions of the second changes yield no clues about what might make *dnaA508 *and *dnaA46 *mutants require *priC *for growth at 30°C and why the other four *dnaA *mutants do not.

What might PriC be doing to help initiation of DNA replication in a *dnaA*^ts ^strain? One idea is that the *dnaA*^ts ^protein is defective in its ability to create a region of ssDNA at *oriC*. Since the RRPs are also thought to help create regions of ssDNA (away from *oriC*) so that DnaC binds and loads DnaB, it is possible that PriC may help the DnaA mutants in this endeavor. Another type of model that is formally possible is that PriC may somehow stabilize the *dnaA*^ts ^protein. This seems unlikely, however, given that *dnaC809*,*820 *can rescue the synthetic lethality of *priC303::kan *and *dnaA508*. Other models may also be possible.

One needs to consider if DnaA may be involved in replication restart. In considering this, one needs to remember that DnaA has the ability to bind DNA at specific sites and bend it. It has been shown that different *dnaA*^ts ^allele can differentially influence the rate of initiation of transcription in some promoters with DnaA binding sites (see below). This in turn can influence replication restart in two ways. First changes in the level of gene expression of a single gene (or groups of genes) may indirectly influence the replication restart process. Second, the ability of DnaA to bind to many sites on the chromosome may influence structure of the nucleoid and the sites at which replication restart may occur.

There are many examples where several phenotypes had been tested systematically for several *dnaA *alleles (Table 1 and [31-33]). The only other system that seems to have some similarity to the data here is one in which the ability to replicate λ *P*^+ ^plasmids was investigated [27,34]. Table 1 shows that *dnaA46*, *dnaA204 *and *dnaA508 *all fail to replicate these plasmids while *dnaA5*, *dnaA167 *and *dna602 *can. The model proposed to explain this phenomenon suggests that DnaA is required to activate transcription at the λ *P*_*R *_promoter and that the *dnaA46*, *dnaA204 *and *dnaA508 *mutations decrease this ability[27]. With the exception of *dnaA204*, the inability to replicate these plasmids mirrors the ability of the *dnaA*^ts ^mutants to grow in the absence of *priC*.

The results presented in this paper do not allow one to definitively know whether the synthetic lethality studied here is due to a failure in initiation of replication at *oriC *or is it due to a failure in replication restart based on the study of the suppressors. Since *dnaA *mutations can affect more than just initiation of DNA replication, it is tempting to speculate that some other *dnaA *function: transcription of a particular gene or set of genes or the shape of nucleoid in the *priC *mutant may contribute or be the cause of the synthetic lethality. Understanding the molecular mechanism underlying the *dnaA*^ts^-*priC *synthetic lethality may require appreciation of these other aspects of *dnaA *biology.

## Conclusions

A hitherto unknown function for either DnaA in replication restart or PriC in initiation of DNA replication that occurs in certain *dnaA *temperature sensitive strains at the permissive temperature of 30°C has been documented. Models considering roles for PriC during initiation of DNA replication and roles for DnaA in replication restart were tested and found not to decisively explain the data. Other roles of *dnaA *in transcription and nucleoid structure are additionally considered.

## Methods

### Bacterial strains

All bacterial strains used in this work are derivatives of *E. coli *K-12 and are described in Table 4. The protocol for P1 transduction has been described elsewhere [37]. All P1 transduction were selected on 2% agar plates containing either minimal or rich media and either tetracycline 10 μg/ml or kanamycin 50 μg/ml final concentration. All transductants were first purified on the same type of media on which they were selected. Tests for temperature sensitivity were then done by replica plating patches of the purified transductants at 30°C and 42°C on solid rich media without any antibiotics. Growth was scored by either the presence or absence of a patch after 24 hours.

### Cloning of the priC gene

Wildtype chromosome DNA was used as the template in a standard PCR reaction using prSJS283 and prSJS284 (Table 3) as the priming oligonulceotides. The amplified PCR fragment (that includes the putative promoter) was purified by gel electrophoresis and cloned into the pCR 2.1 using the TOPO-TA cloning system from Invitrogen. The *priC *containing plasmid was called pTH1.

## Authors contributions

TH carried out the initial part of the molecular genetic studies. These were completed by SJS. SJS conceived of the study and wrote the manuscript.

## Figures and Tables

**Table 1:** *dnaA *alleles used in this work and some phenotypes

Allele	Amino Acid Change	Domain Affected^*a*^	Ability to replicate P^+ ^plasmids at 30°C^*b*^	Ability to grow in *priC303::kan *at 30°C
*dnaA46*	A184V H252Y	III III	No	No
*dnaA508*	P28L T80I	I I	No	No
*dnaA5*	A184V G426S	III IV	Yes	Yes
*dnaA167*	V157E	III	Yes	Yes
*dnaA204*	I389N	IV	No	Yes
*dnaA602*	A184V A347V	III linker	Yes	Yes

^*a*^Domains are defined according to [25, 35, 36].

^*b *^Data from [34]

**Table 2:** P1 crosses using *dnaA*^ts ^strains as the donors and isogenic *priC303::kan *and *dnaC *mutants as the recipients^*a*^

Donor		Recipient Strain Number	Recipient Genotype		Ts/total
Strain Number	*dnaA*		*priC*	*dnaC*	
JC12390	*508*	JC13509	*+*	*+*	49/53
AL0454	*46*	JC13509	*+*	*+*	60/69
JC12390	*508*	SS145	*303*	*+*	0/72
AL0454	*46*	SS145	*303*	*+*	0/72
SS1750	*5*	SS145	*303*	*+*	7/12
SS1751	*167*	SS145	*303*	*+*	11/11
WM433	*204*	SS145	*303*	*+*	4/5
SS1752	*602*	SS145	*303*	*+*	10/12
JC12390	*508*	SS1099	*303*	*809*	0/63
JC12390	*508*	SS1100	*303*	*809,820*	41/48
AL0454	*46*	SS1099	*303*	*809*	0/32
AL0454	*46*	SS1100	*303*	*809,820*	0/24

^a^These are the results of either single or multiple transductions. The numbers show the number of Tet^R ^transductants that were also sensitive to growth at 42°C. If the transductions yielded temperature sensitive transductants then only a few transductants from one transduction is reported. The recipient cells were grown at 37°C in Luria broth. They were then treated with donor lysate made on the *tnaA300::*Tn*10 dnaA*^ts ^strain indicated. The transductions were then spread on Luria plates containing 10 μgm/ml tetracycline and incubated 24–48 hours at 30°C. Transductants were then picked, purified and tested for growth at 42°C by replica plating.

**Table 3:** List of Oligonucleotide Primers

Name	5' to 3' oligonucleotide sequence	Position
prSJS283	ATATTGAGTGTTGTCAGC	Upstream of *priC*
prSJS284	TCCTCCAGCAGCACAATC	Downstream of *priC*
prSJS480	CCGCGGTCCCGATCGTTTTG	*dnaA *upstream primer
prSJS481	GCAGGGCGTTGAAGGTGTGG	*dnaA *downstream primer

**Table 4:** Strain List

Strain	*priC*	*dnaA*	*dnaC*	Relevant Genotype	Source or Derivation
ALO454	*+*	*46*	*+*	*tnaA300::Tn10*	Kirsten Skarstad
AQ12251	*+*	*+*	*+*	*rnhA339::cat*	T. Kogoma
CAG18442	*+*	*+*	*+*	*thr-34::Tn10*	[38]
CM740	*+*	*5*	*+*		Kirsten Skarstad
CM2556	*+*	*167*	*+*		Kirsten Skarstad
CM2733	*+*	*602*	*+*		Kirsten Skarstad
JC12390	*+*	*508*	*+*	*tnaA300::Tn10*	Lab stock
JC13509^*a*^	*+*	*+*	*+*		[18]
JC19008	*+*	*+*	*809*	*priA2::kan*	[28]
JC19165	*303*	*+*	*+*		[18]
JC19257	*+*	*+*	*809,820*	*priA2::kan*	[18]
SS145	*303*	*+*	*+*		JC19165 -> JC13509^*b*^
SS1091	*+*	*+*	*809*		JC19008 -> SS1213^*c*^
SS1092	*+*	*+*	*809,820*		JC19257 -> SS1213^*c*^
SS1099	*303*	*+*	*809*		JC19165 -> SS1091^*b*^
SS1100	*303*	*+*	*809,820*		JC19165 -> SS1092^*b*^
SS1201	*+*	*508*	*+*	*tnaA300::Tn10*	JC12390 -> JC13509^*e*^
SS1213	*+*	*+*	*+*	*thr-34::Tn10*	CAG18442 -> JC13509^*d*^
SS1504	*303*	*508*	*809,820*	*tnaA300::Tn10*	JC12390 -> SS1100^*e*^
SS1531	*303*	*+*	*+*	*rnhA339::cat*	AQ12251 -> SS145
SS1543	*303*	*508*	*+*	*rnhA339::cat*	JC12390 -> SS1531^*f*^
SS1738	*+*	*+*	*+*	*tnaA300::Tn10*	Lab Stock
SS1750	*+*	*5*	*+*	*tnaA300::Tn10*	SS1738 -> CM740^*e*^
SS1751	*+*	*167*	*+*	*tnaA300::Tn10*	SS1738 -> CM2556^*e*^
SS1752	*+*	*602*	*+*	*tnaA300::Tn10*	SS1738 -> CM2733^*e*^
SS1793	*303*	*167*	*+*	*tnaA300::Tn10*	SS1751 -> SS145^*e*^
SS1796	*303*	*602*	*+*	*tnaA300::Tn10*	SS1752 -> SS145^*e*^
SS1797	*303*	*5*	*+*	*tnaA300::Tn10*	SS1750 -> SS145^*e*^
SS1798	*303*	*204*	*+*	*tnaA300::Tn10*	WM433 -> SS145^*e*^
SS3032	*303*	*46*	*+*	*rnhA339::cat*	ALO454 -> SS1531^*f*^
WM433	*+*	*204*	*+*	*tnaA300::Tn10*	Mark Sutton

^*a *^JC13509 has the following genotype: *sulB103 lacMS286 Φ 80dIIlacBK1 argE3 his-4 thi-1 xyl-5 mtl-1 *Sm^R ^T6^R^. The *lacMS286 Φ 80dIIlacBK1 *code for two partial copies of the *lac *operon [39, 40].

^b^Select for kanamycin resistance on minimal media.

^c^Select for threonine prototrophy on minimal media. Screen for the presence of the *dnaC *mutation by the method described in [18].

^d^Select for tetracycline resistance on rich media and screen for threonine auxotrophy.

^e^Select for tetracycline resistance on rich media at 30°C and then screen for temperature sensitive growth at 42°C.

^f^Select for tetracycline resistance on rich media at 30°C and then screen for restriction site polymorphism and or backcross to test the presence of the temperature sensitive allele.
